# Genotype-Specific Plastic Responses to Seed Bacteria under Drought Stress in *Lactuca serriola*

**DOI:** 10.3390/microorganisms10081604

**Published:** 2022-08-09

**Authors:** Seorin Jeong, Tae-Min Kim, Byungwook Choi, Yousuk Kim, Hwan Kim, Eunsuk Kim

**Affiliations:** 1 School of Earth Sciences and Environmental Engineering, Gwangju Institute of Science and Technology, Gwangju 61005, Korea; 2 GIST Central Research Facilities, Bio Imaging Laboratory, Gwangju Institute of Science and Technology, Gwangju 61005, Korea

**Keywords:** drought tolerance, invasive plants, plant genotypes, seed bacteria, soil water contents

## Abstract

Recent studies have demonstrated that seed-borne bacteria can enhance the performance of invasive plants in novel introduced habitats with environmental stresses. The effect of this plant-bacteria interaction may vary with plant species or even genotype; however, the genotype-dependent effects of seed bacteria have rarely been assessed. In this study, we examined the effects of bacterial strains isolated from seeds on the genotypes of an invasive xerophytic plant, *Lactuca serriola*. Plant genotypes were grown under drought conditions, and their plastic responses to bacterial infections were evaluated. Some genotypes produced more biomass, whereas others produced less biomass in response to infection with the same bacterial strain. Notably, the quantity of root-adhering soil depended on the bacterial treatment and plant genotypes and was positively correlated with the plastic responses of plant performance. Because tested bacteria could colonize the plant rhizosphere, bacterial infection appears to induce the differential formation of soil rhizosheaths among plant genotypes, consequently affecting the maintenance of soil water content under drought conditions. Given that drought tolerance is a critical attribute for the invasive success of *L. serriola*, these results imply that bacterial symbionts can facilitate the establishment of alien plant species, but their effects are likely genotype-specific.

## 1. Introduction

Plant-associated microbes have been suggested to constitute plant holobiomes and influence plant development and growth [1,2]. Plants can benefit from microbes that assist in nutrient acquisition or produce exogenous phytohormones [3,4]. In addition, plant growth-promoting (PGP) microbes can protect the host plant from abiotic stresses by diverse stress ameliorating mechanisms [5,6].

Recent studies have proposed that PGP microbes can contribute to the invasive capability of exotic plant species [7,8,9]. They can alleviate biotic and abiotic stresses in newly arrived habitats, and consequently, stimulate plant establishment and competitive ability. Among plant-associated microbes, endophytic bacteria residing inside the seeds are of particular interest. Because seeds and microbes would move together to the new habitat, seed bacteria can provide an advantage to invasive plants regardless of the existence of mutualistic microbes in the introduced sites [10]. In addition, seed endophytes are expected to be transmitted vertically to their offspring; hence, their effects can be present for generations [11].

A xerophytic invasive plant, *Lactuca serriola* (Asteraceae), has rapidly expanded its geographic range in South Korea since its first report in 1978 [12]. In a previous study, bacteria were isolated from the seeds of *L. serriola,* and their drought stress-ameliorating effects were evaluated in a model plant species, *Arabidopsis thaliana* [10]. The bacterial isolates grew vigorously under high osmotic pressure, indicating that they could tolerate drought stress. One strain out of the 12 isolates, *Kosakonia cowanii* GG1, stimulated the growth of *A. thaliana* under drought stress. However, the mutualistic interaction between plants and bacteria tends to depend on the host plant species [13,14]. Thus, the effects of isolated bacteria on plant performance should be examined in *L. serriola* host plants to evaluate their ecological significance in terms of invasiveness.

Bacteria producing exopolysaccharides have been demonstrated to alter soil characteristics, consequently ameliorating drought stress. Bacterial exopolysaccharides can contribute to soil aggregation and water retention around plant roots [10,15]. In addition, exopolysaccharides can induce a firm adherence of soil to the roots, which is referred to as the rhizosheath. A thick rhizosheath can alleviate drought stress by securing soil water [16,17]. In *A. thaliana*, *K. cowanii* GG1 can colonize in the root and produce a relatively high amount of exopolysaccharides when grown in media with a negative water potential. Based on these observations, the production of exopolysaccharide by *K. cowanii* GG1 was suggested to promote the drought tolerance of *A. thaliana*. A similar mechanism might occur in *L. serriola*.

Similar to species-specific responses to endophytic bacteria, plant genotypes may also respond differently to endophytic bacteria. The role of plant genotypes in shaping seed microbiome has been well-established, suggesting that plant-endophyte interaction is possibly dependent on plant genotypes [18,19]. However, few studies explicitly assessed the responses of multiple plant genotypes to bacterial treatments and the information about genotype-specificity is still inconclusive [20,21,22]. From an evolutionary ecological perspective, characterizing plant genetic variation in response to endophytic bacteria is of interest because the variation indicates that the fitness difference among plant genotypes probably depends on the existence of endophytic bacteria.

In this study, we inoculated the previously isolated seed endophytic bacteria into their original host, *L. serriola*, and evaluated whether the isolates alleviated drought stress in the host plant. In particular, we assessed the plastic responses of five maternal genotypes of *L. serriola* to bacterial infection under drought stress to examine genotype-specific responses to bacterial treatments. Bacterial strains transformed with a plasmid containing green fluorescent protein (GFP) gene were used to confirm colonization by the bacterial isolates. Effects of bacterial inoculation on soil characteristics were also assessed, including the quantity of root-adhering soil and its polysaccharide content. 

Specifically, we addressed the following questions: (1) Do the genotypes of *L. serriola* exhibit differential plastic responses to the infection of seed bacteria under drought conditions? (2) Do the infection of seed bacteria and plant genotypes influence soil characteristics? (3) Does the plant performance under drought conditions correlate with soil characteristics? 

## 2. Materials and Methods

### 2.1. Preparation of Isolates Transformed with a Plasmid Containing GFP for the Infection into Seeds

To confirm whether the isolates colonized the plants, ten isolates (*Pantoea septica* YJ1, *Kosakonia cowanii* YJ4, *Erwinia tasmaniensis* YJ6, *K. cowanii* SD1, *P. ananatis* SD16, *P. dispersa* SD25, *Cronobacter dublinensis* subsp. *dublinensis* MS24, *C. dublienesis* subsp. *lausanneisis* MS26, *K. cowanii* GG1, and *P. ananatis* GG19) from a previous study were subjected to transformation with a broad host range plasmid pDSK-GFPuv (#PVT12964, Nova Lifetech, Singapore) containing GFPuv protein and kanamycin resistance gene for selection [10,23]. The plasmid was amplified using chemically competent *Escherichia coli* DH5a (#CP011, Enzynomics, Daegeon, Korea) cells that were transformed using the heat-shock method and then extracted from the cells using the QIAprep Spin Miniprep Kit (#27106, Qiagen, Hilden, Germany). Electrocompetent cells were prepared following the manufacturer’s instructions using a MicroPulser (#165-2100, Bio-Rad Laboratories, Hercules, CA, USA). The pDSK-GFPuv plasmid (2 µL) was injected into 40 µL of electrocompetent cell suspension using a MicroPulser with a 0.1 cm electroporation cuvette (#165-2089, Bio-Rad). A pulse of 1.8 kV voltage at 5 ms intervals was applied. After recovery in Luria–Bertani (LB) broth (#244620, BD Difco, Franklin Lakes, NJ, USA) at 37 °C for 1 h, the transformed colonies were selected on LB agar (#244520, BD Difco) medium containing kanamycin (50 µg/mL) and stored at −80 °C. 

### 2.2. Experimental Design

The seeds of five maternal genotypes of *L. serriola* collected from Bongsan-ro, Cheongju-si, South Korea (latitude: 36.620556, longitude: 127.328611), were used. Seed surfaces were sterilized by soaking the seeds in 3% sodium hydrochloride for 3 min. Seeds were inoculated with the ten isolates by immersing them overnight in a bacterial suspension (10^8^ CFU mL^−1^) containing phosphate-buffered saline (PBS) [24]. Seeds submerged in PBS were used as controls. The GFP-transformed isolates were used to confirm bacterial infection after harvesting.

Seeds were sown in trays with 105 holes with sterile commercial soil and watered daily. Twenty-five days after sowing, six seedlings of each maternal genotype were randomly chosen in each bacterial treatment. A single seedling was transplanted into a pot (8 cm × 7.5 cm × 6 cm) containing sterile vermiculate (Green Fire Chemicals, Hongseong, Korea) and sand (Glpark, Seoul, Korea) in a ratio of 3:1 (volume) [25]. Because most of the seeds inoculated with *P. ananatis* SD16, *P. dispersa* SD25, and *C. dublinensis* subsp. *dublinensis* MS24 failed to germinate, they were excluded from further experiments. This resulted in a total of 240 seedlings for the drought treatment (eight bacterial treatments including negative control × five maternal genotypes × six replicates).

The pots were placed in a walk-in chamber, following a completely randomized design, maintained at 22 °C under a 12/12 h light/dark photoperiod throughout the experiment. Deionized water was applied on alternative days, and the Hoagland’s solution (1.25 mM KNO_3_, 1.5 mM Ca(NO_3_)_2_, 0.75 mM MgSO_4_, 0.5 mM KH_2_PO_4_, 0.05 mM H_3_BO_3_, 0.01 mM MnCl_2_, 0.002 mM ZnSO_4_, 0.0015 mM CuSO_4_, 0.075 μM NH_4_Mo_7_O_24_, and 0.074 mM Fe-EDTA) was applied once a week to ensure nutrient supply [25]. Drought treatment was applied 42 d after sowing and maintained for 13 days. Among the six plants of each maternal genotype in each bacterial treatment, four plants were subjected to drought conditions by stopping their water supply, and the remaining two plants were watered regularly to confirm the effects of the drought treatment. Five individuals in the uninoculated control accidentally died during the maintenance period of transplanted seedlings and drought treatment. Thus, two plants were watered regularly, and two to four plants were imposed under drought conditions in each maternal genotype and each bacterial treatment. The sample size for each bacterial treatment and plant genotype is given in Table S1. 

### 2.3. Plant Trait Measurements

After 13 d of drought treatment, the plants were harvested for trait measurements. The shoot and root fresh weights were measured immediately after harvesting. The roots were dried in a drying oven (Hanbaek Co. Ltd., Anyang-si, Kyunggi-do, Korea) at 70 °C for 72 h for dry weight measurements [26]. To confirm the colonization of the infected isolates in the plant rhizosphere, five out of twenty plants in each bacterial treatment under drought stress were randomly selected. Root tissues of these plants were pooled together, cut into small pieces using sterilized scissors, and placed on LB agar medium containing kanamycin (50 µg/mL). The plates were incubated at 28 °C, and GFPuv-expressing bacterial colonies were observed under UV light.

To evaluate the effects of bacterial treatment on the physiological responses of plants to drought stress, we measured the relative water content (RWC) and malondialdehyde (MDA) content in the leaves. In response to drought, the RWC of plants has been known to decrease and the MDA content in leaves has been known to increase due to oxidative damage to membrane lipids. The RWC in one leaf was determined by calculating the difference between the fresh weight and dry weight, which was measured after drying the leaf in a drying oven at 70 °C for 72 h, divided by the difference between the fresh weight and turgid weight, which was measured after submerging the leaf in deionized water for 24 h at 4 °C [27]. The MDA content in leaf tissue (0.1 g), which was stored at −80 °C immediately after harvesting, was measured using thiobarbituric acid reactive substances (TBARS) assay [28,29]. The leaves were ground using TissueLyser II (QIAGEN) and homogenized using 1 mL of 0.1% (*w*/*v*) trichloroacetic acid (TCA, T6399, Sigma-Aldrich, St. Louis, MI, USA). After centrifugation, the supernatant was mixed with 4 mL of 20% TCA containing 0.5% thiobarbituric acid (T5500, Sigma-Aldrich), and the mixture was boiled at 95 °C for 15 min. The absorbance of the solution was measured at 532 nm using the BioSpectrometer basic (Eppendorf, Hamburg, Germany).

### 2.4. Soil Characteristics

Three soil characteristics were examined to determine the bacterial effects. The gravimetric soil water content was measured as the difference between the weight of the soil at harvest and the weight of the soil after drying at 105 °C for 72 h. Root-adhering soil (RAS) was collected in a conical tube by washing the roots with ddH_2_O after gentle shaking and air-drying. The soil (5 mg) was transferred to another tube, and the concentration of soil polysaccharides was measured using the direct determination method as previously described [30]. The dry weight of the RAS was measured after drying at 105 °C for 72 h. 

### 2.5. Visualization of GFP-Transformed Isolates Using Confocal Microscopy

Seeds of genotypes one (P1) and five (P5) were infected with GFPuv-transformed bacterial isolates as described previously and then sown on Murashing and Skoog medium (M0222.0050, DUCHEFA, Haarlem, The Netherlands) containing 1.5% Bacto Agar (214010, BD Difco). After germination, the roots of seedlings were fixed with 4% paraformaldehyde (158127, Sigma-Aldrich) for 120 min, and then washed twice with PBS solution [31]. To eliminate autofluorescence from plant tissue, the samples were soaked in ClearSee solution (10 % (*w*/*v*) xylitol powder, 15 % (*w*/*v*) sodium deoxycholate, 25 % (*w*/*v*) urea; 1.410 refractive index) for 1 week. Thereafter, the samples were stained using calcofluor white (18909, Sigma-Aldrich) for 30 min, followed by washing with the ClearSee solution for 30 min [32]. The samples were observed under a FV3000RS confocal laser scanning microscope (CLSM) with a UPLSAPO 30× silicon lens (Olympus, Tokyo, Japan). The images were taken using the z-stack method at 0.620 µm intervals. Calcofluor white signal was measured at 461 nm emission with 405 nm excitation, whereas the GFPuv signal was measured at 513 nm emission with 488 nm excitation.

### 2.6. Statistical Analyses

All statistical analyses were performed using R software 4.0.1 (R Foundation for Statistical Computing, Austria). A mixed-model analysis of covariance (ANCOVA) was performed to confirm the effect of drought treatment on plant performance. Drought treatment was a fixed factor, and maternal genotype and bacterial treatment were random factors. Leaf length at transplanting was included as a covariate to control the influence of plant size on the effect of drought treatment.

A subset of the entire dataset, including pots with drought treatment, was constructed to examine plant responses to bacterial treatments under drought conditions. The relative distance plasticity index (RDPI) is well-acknowledged as an appropriate measure for comparing differential plastic responses to treatments among plant genotypes [33]. We adopted this method and calculated the RDPI of the bacterial treatments for each plant genotype. First, the relative distances of trait values were calculated as differences in trait values divided by the sum of trait values between all pairs of individuals in the different bacterial treatments. The RDPI of the bacterial treatment was measured as the average of the relative distances between the control and each bacterial infection. For calculating the relative distance, the difference in trait values was used instead of the absolute values of the difference to consider the direction of plastic responses. The number of data points ranged from eight to sixteen for each bacterial treatment and each plant genotype. 

Two-way analyses of variance were conducted to compare the RDPI of bacterial treatments among plant genotypes. The model comprised bacterial treatment, plant genotype, and their interactions as fixed factors. Tukey’s post hoc multiple comparison tests were conducted for each bacterial treatment to interpret the interactions between bacteria and plant genotypes.

## 3. Results

**Figure 1:**
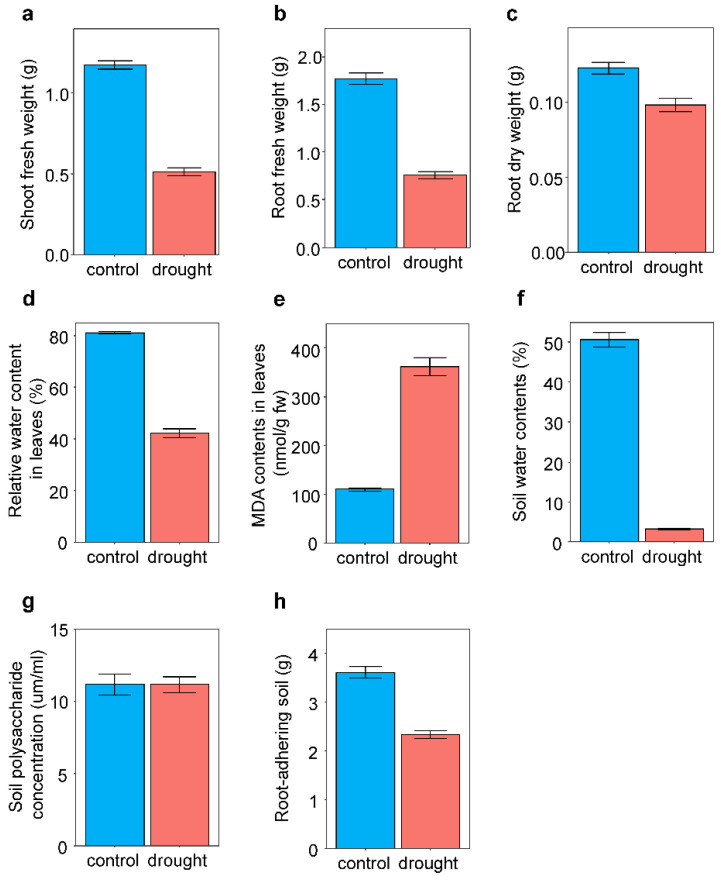
Effects of drought treatment on plant traits and soil characteristics Average values and standard errors of (**a**) shoot fresh weight, (**b**) root fresh weight, (**c**) root dry weight, (**d**) relative water content in leaves, (**e**) MDA contents in leaves, (**f**) soil water contents, (**g**) soil polysaccharide concentration, and (**h**) root-adhering soil are given.

In response to drought treatment, plants exhibited reduced shoot (F_1,309_ = 476.15, *p* < 0.001) and root (F_1,310_ = 336.68, *p* < 0.001) fresh weights and root dry weight (F_1,262_ = 27.73, *p* < 0.001) compared with those in the control (Figure 1). RWC in leaves decreased (F_1,318_ = 242.38, *p* < 0.001) and MDA content in leaves increased (F_1,317_ = 275.53, *p* < 0.001) in response to the drought treatments (Figure 1). Drought treatment decreased the soil water content by at least 16%.

**Figure 2:**
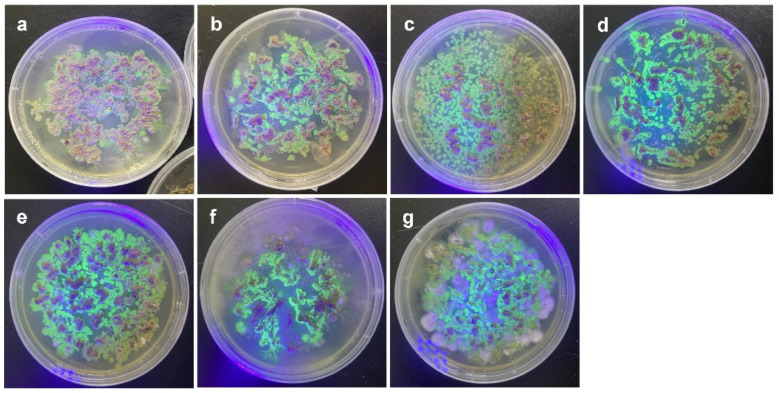
Observation of GFPuv-expressing colonies isolated from bacteria-treated root tissues under UV lamp. (**a**) *Pantoea septica* YJ1, (**b**) *Kosakonia cowanii* YJ4, (**c**) *Erwinia tasmaniensis* YJ6, (**d**) *K. cowanii* SD1, (**e**) *C. dublienesis* subsp. *lausanneisis* MS26, (**f**) *K. cowanii* GG1, (**g**) *P. ananatis* GG19.

**Figure 3:**
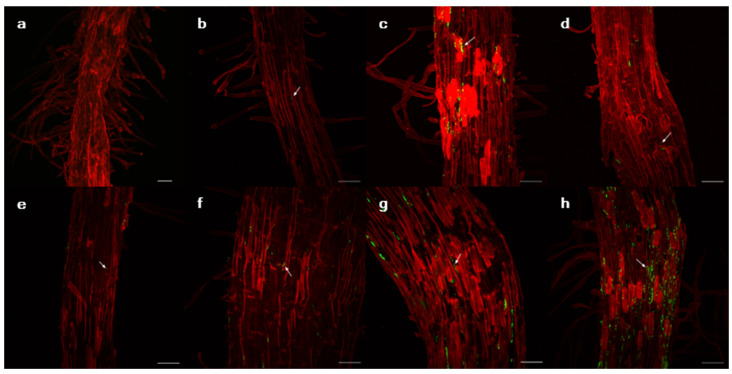
Images of the root tissues of *Lactuca serriola* genotype P1 with GFPuv-transformed isolates using confocal microscopy. The plant cell wall and isolates are indicated by red and green, respectively. (**a**) Uninfected control, (**b**) *Pantoea septica* YJ1, (**c**) *Kosakonia cowanii* YJ4, (**d**) *Erwinia tasmaniensis* YJ6, (**e**) *K. cowanii* SD1, (**f**) *C. dublienesis* subsp. *lausanneisis* MS26, (**g**) *K. cowanii* GG1, (**h**) *P. ananatis* GG19. Scale bar: 50 µm.

Bacterial colonies with fluorescence were observed when the roots of the plants were incubated on agar plates (Figure 2). When two plant genotypes (P1 and P5) were infected by the bacterial strains, colonized bacteria were observed inside and on the surface of the roots (Figure 3 and Figure S1). 

**Figure 4:**
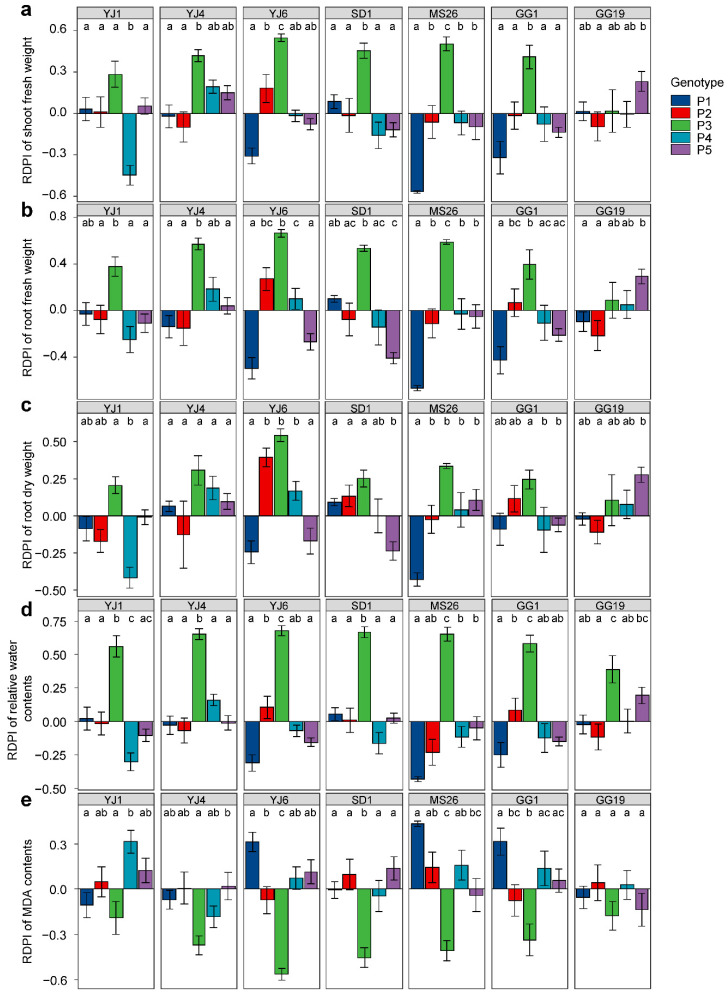
Plastic responses of different *Lactuca serriola* genotypes to bacterial treatments. Average relative distance plasticity indexes (RDPIs) and standard errors of (**a**) shoot fresh weight, (**b**) root fresh weight, (**c**) root dry weight, (**d**) relative water contents, and (**e**) MDA contents are presented. Letters indicate statistically significant (*p* < 0.05) differences among genotypes based on Tukey’s multiple comparison tests. The names of bacterial strains are given in Figure 1.

*L. serriola* genotypes exhibited differential plastic responses to bacterial treatments for all measured plant traits when grown under drought conditions, as indicated by the significant interactions between the genotype and bacterial treatment (Table 1). Plant genotype P3 exhibited positive RDPIs for shoot fresh weight, root fresh weight, and root dry weight in response to all bacterial treatments, except *P. ananatis* GG19 (Figure 4). In addition, genotypes P2, P4, and P5 showed positive RDPIs with *E. tasmaniensis* YJ6, *K. cowanii* YJ4, and *P. ananatis* GG19, respectively, for the shoot and root biomass (Figure 4). The positive RDPIs indicate that plants infected by bacterial isolates produced higher shoot and root biomass than the control plants. In contrast, negative RDPIs of root and shoot biomass were found for the P1 and P5 genotypes infected by *E. tasmaniensis* YJ6, *C. dublienesis* MS26, *K. cowanii* SD1, or *K. cowanii* GG1, indicating that these plants produced less biomass than the control plants under drought conditions (Figure 4). Bacterial treatments that resulted in positive RDPIs for plant biomass tended to cause positive RDPIs for leaf RWC and negative RDPIs for leaf MDA content (Figure 4). The positive RDPIs for MDA content indicate that drought stress increased the oxidative damage to membrane lipids.

**Figure 5:**
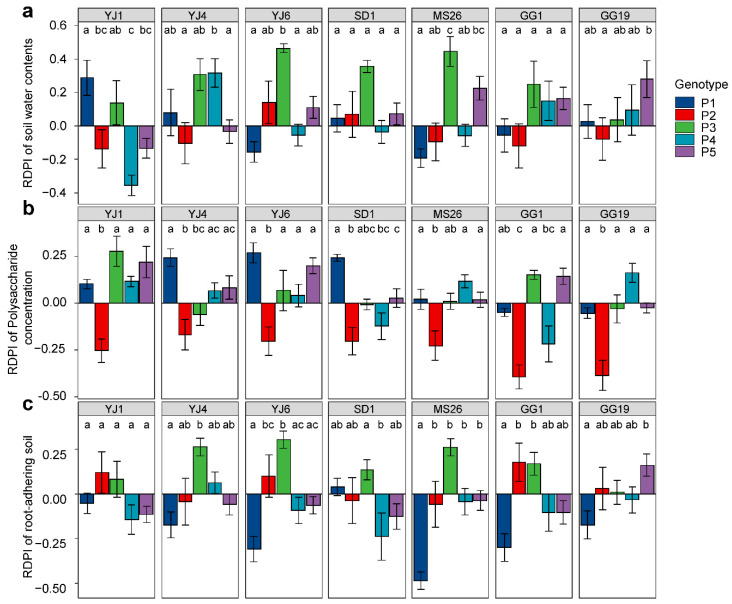
Plastic responses of different *Lactuca serriola* genotypes to bacterial treatments. Average relative distance plasticity indexes (RDPIs) and standard errors of (**a**) soil water contents, (**b**) soil polysaccharide concentration, and (**c**) root-adhering soil are presented. Letters indicate statistically significant (*p* < 0.05) differences among genotypes based on Tukey’s multiple comparison tests. The names of bacterial strains are given in Figure 1.

**Figure 6:**
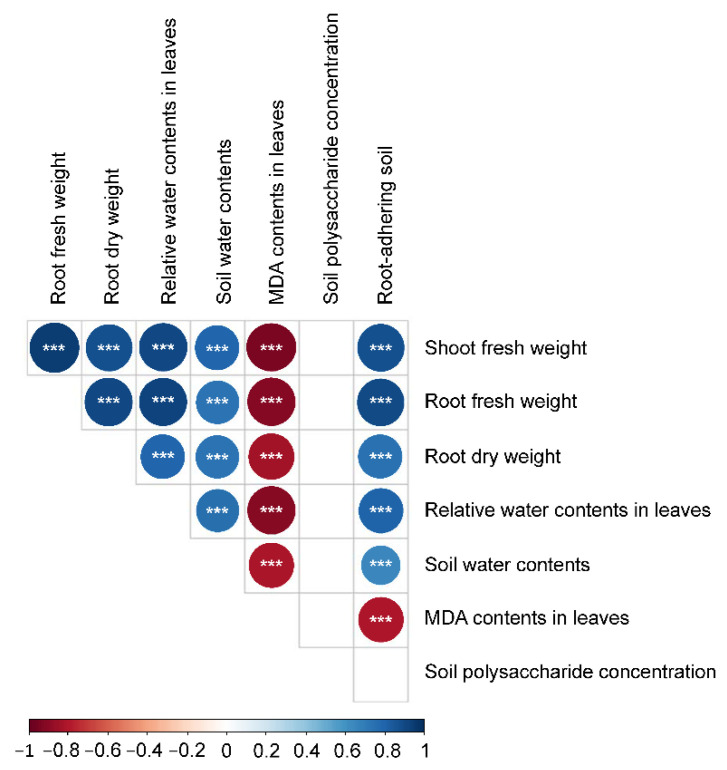
Correlogram of Spearman’s correlation coefficients between RDPIs of plant traits and soil characteristics. Negative correlations are in red and positive correlations are in blue. *** *p* < 0.001.

The RDPIs for the measured soil characteristics exhibited a pattern similar to that of the RDPIs for plant biomass. Pot soil with plants exhibiting positive RDPIs for biomass tended to have positive RDPIs for RAS and soil water content (Figure 5). In addition, the RDPIs for soil water, RAS, and plant biomass production were positively correlated as determined by the Pearson’s correlation analysis (Figure 6). The RDPIs for polysaccharide concentration were not correlated with the RDPIs for the other measured traits.

## 4. Discussion

Bacterial strains isolated from the seeds of invasive *L. serriola* could colonize the rhizosphere of their original host. When plants were grown under drought conditions, plant genotypes exhibited differential plastic responses to bacterial treatments, indicating that the effects of bacterial infection on plant performance depend on the plant genotype. 

### 4.1. Genotype-Specific Responses to Bacterial Infection

In plant genotype P3, six out of seven tested bacterial strains stimulated the shoot and root biomass of plants under drought stress. They increased RWC and reduced MDA content in leaves, indicating that they ameliorated the drought stress imposed on plants. Similar to this study, inoculation of the endophytic bacterium *C. dublinensis* strain MKS-1 in pearl millet increased plant biomass, leaf RWC, and osmotolerant proline content under drought stress [34]. Additionally, although the drought-tolerant effects of other species are unknown, the salt stress-ameliorating effects of *K. cowanii* and *P. ananatis* have been reported previously [35,36].

Notably, the beneficial effects of seed bacteria were manifested mostly in genotype P3, and the same bacterial strains had neutral or even negative effects on the growth of other plant genotypes. Previous studies have reported contrasting results regarding the effects of plant genotypes. For instance, *A. thaliana* accessions showed variable responses in shoot and root development when applying volatile organic compounds produced by *Pseudomonas simiae* [37]. Additionally, wheat cultivars infected with *Paenibacillus* sp. show differential resistance to pathogens [20]. In contrast, *Pseudomonas* sp. and *Methylorubrum* sp. isolated from rice seeds show similar growth-promoting activities in the two rice cultivars [21]. Our results support the hypothesis that the effects of endophytic bacteria depend on the plant genotype. 

Suppose that drought tolerance is one of the critical attributes for the invasive success of *L. serriola*. In that case, seed bacteria can facilitate the invasion of some genotypes by promoting plant performance under drought conditions, but they can also hinder the invasion of other genotypes. This suggests that the interaction between plants and seed bacteria should be assessed with respect to the genotype of host plants to evaluate the ecological significance of seed bacteria. Given the diverse effects on plant performance [1,3,11], it is questionable whether bacterial strains with adverse impacts on the plant performance in dry conditions also have negative effects in different environmental conditions. Examining plant-microbe interactions in diverse environmental conditions is required for a more complete understanding of the role of seed bacteria in invasive success.

### 4.2. Effects of Bacterial Infection on Rhizosphere Characteristics

Isolates that were used to infect *L. serriola* seeds successfully colonized the rhizosphere, as confirmed by the isolation of GFPuv-expressing colonies from root tissue and microscopic observation. The confocal images showed that the isolates colonized both the root surface and endosphere, indicating that the isolates also colonized the surrounding environments [38]. These bacteria can potentially manipulate the rhizosphere microenvironment.

In this study, the RDPI for soil water content was positively correlated with the RDPI for plant biomass and RAS quantity across bacterial treatments and plant genotypes. Given that a thick rhizosheath can ameliorate drought stress [16,17], the bacterial infection and plant genotype might affect the RAS quantity together, consequently influencing soil water content and biomass production under drought conditions. 

The quantity of RAS is highly dependent on root hairs and adhesive mucilage, of which polysaccharides constitute the major component [39]. Bacteria in the rhizosphere can produce exopolysaccharides for their own protection in extreme environments [22]. As a side effect, extracellular polysaccharides can aggregate soil particles, increase the amount of RAS, and facilitate water retention under drought conditions [40]. *P. ananatis* GG19 and *K. cowanii* YJ4 and SD1 strains can produce more exopolysaccharides when exposed to high osmotic pressure [10]. When they colonized the rhizosphere of the P3 genotype plants, their exopolysaccharide production might have contributed to the formation of RAS and induced higher drought tolerance. 

Suppose that bacterial production of exopolysaccharides is a major mechanism for promoting RAS formation. In that case, the degree of bacterial colonization on the root surface is expected to correlate with the RAS quantity. Although the colonization ability could not be measured quantitatively, the confocal microscopy suggested that *K. cowanii* GG1 and *P. ananatis* GG19 seemed to colonize in the root surface of the P1 genotype more than other testing strains (Figure 3). Notably, contrasting plastic responses of plants to those two strains were observed. The P1 genotype rarely responded to the infection of *P. ananatis* GG19, but it exhibited negative RDPI of the biomass production in response to *K. cowanii* GG1, similar to plants infected by *C. dublienesis* MS26 (Figure 4). This result suggests that factors other than exopolysaccharide production likely contribute to the RAS production.

Bacterial production of phytohormones can influence RAS quantity. All tested bacterial strains, except *K. cowanii* GG1, can produce both indole acetic acid and 1-aminocyclopropane-1-carboxylate (ACC) deaminase, which can modulate the levels of phytohormones [10]. Production of indole acetic acid by plant-associated bacteria can stimulate root hair development [41], which possibly increases the RAS quantity. Furthermore, reduced ethylene levels by ACC deaminase can promote the production of RAS [42]. 

In addition, the inoculated bacteria themselves might not be involved in the changes in soil characteristics. Instead, inoculated bacteria might alter the microbial community in the rhizosphere, which might stimulate the formation of RAS and increase soil water contents. For instance, applying the *Paenibacillus* strain to lettuce plants induced the resistance to soil pathogen not directly but indirectly by shifting microbial community in the rhizosphere [43]. In a similar way, inoculated bacteria might increase other bacteria in the rhizosphere that have the ability to alleviate drought stress. Next-generation sequencing of root endosphere and rhizosphere can provide information to test this hypothesis [43].

The colonization and functional traits of plant-associated rhizobacteria are widely known to be affected by the composition of root exudates from the host plants [44,45,46]. Because the bacterial strains in this study colonized the rhizosphere (Figure 3 and Figure S1), their genotype-specific effects might be due to the difference in root exudate compounds among plant genotypes. Root exudates can regulate gene expression of plant-associated bacteria related to diverse metabolic pathways and stress responses [47]. Variation in root exudates might modulate the production of exopolysaccharides and phytohormones in testing bacterial strains or other bacteria in the rhizosphere, which might induce genotype-specific biomass production under drought conditions. Transcriptome analysis of the host plant and isolates can provide more information about the stress ameliorating mechanism of isolates [48,49].

In conclusion, bacteria isolated from the seeds of *L. serriola* can exert beneficial effects on their origin under drought stress; however, the effects depend on the plant genotype. Both plant genotype and bacterial strain influenced the quantity of root-adhering soil, correlating with plant performance and soil water contents. These results imply that plant genotype is a determining factor in the contribution of symbiotic seed bacteria to drought tolerance, a critical attribute for the invasive success of *L. serriola*.

## Figures and Tables

**Table 1:** Results of analysis of variance comparing relative distance plasticity index (RDPI) among plant genotypes in response to bacterial treatments under drought conditions. F ratios are given. * *p* < 0.05, ** *p* < 0.01, *** *p* < 0.001.

	Genotype (d.f. = 4)	Bacterial treatment (d.f. = 6)	Genotype × Bacteria (d.f. = 24)
RDPI of plant traits			
Shoot fresh weight	9.30 ***	8.82 ***	4.82 ***
Root fresh weight	4.12 **	7.75 ***	4.96 ***
Root dry weight	5.30 ***	6.15 ***	4.97 ***
Relative water contents in leaves	15.45 ***	7.07 ***	3.70 ***
MDA contents in leaves	4.24 **	6.23 ***	2.70 ***
RDPI of soil characteristics			
Soil water contents	6.62 ***	2.79 *	3.00 ***
Root-adhering soil	1.90	4.40 ***	2.25 ***
Polysaccharide concentration	11.87 ***	5.78 ***	3.43 ***

## Data Availability

All data is included in the manuscript.

## Supplementary Materials

The following supporting information can be downloaded at: https://www.mdpi.com/article/10.3390/microorganisms10081604/s1, Figure S1: Images of the root tissues of *Lactuca serriola* genotype P5 with GFPuv-transformed isolates using confocal microscopy. **Table S1**. Sample size for each bacterial treatment and each plant genotype.
