# Supplementary material for: Genotype-Specific Plastic Responses to Seed Bacteria under Drought Stress in Lactuca serriola
Source: Microorganisms. 2022 Aug 9;10(8):1604. doi: 10.3390/microorganisms10081604 (PMC9415285; doi:10.3390/microorganisms10081604)
Supplement: Supplementary file 1 [file microorganisms-10-01604-s001.zip › microorganisms-1821256-supplementary.pdf]

Figure S1. Images of the root tissues of *Lactuca serriola* genotype P5 with GFPuv-transformed isolates using confocal microscopy. The plant cell wall and isolates are indicated by red and green, respectively. (a) Uninfected control, (b) *Pantoea septica* YJ1, (c) *Kosakonia cowanii* YJ4, (d) *Erwinia tasmaniensis* YJ6, (e) *K. cowanii* SD1, (f) *C. dublienesis* subsp. *lausanneisis* MS26, (g) *K. cowanii* GG1, (h) *P. ananatis* GG19. Scale bar: 50 μm.

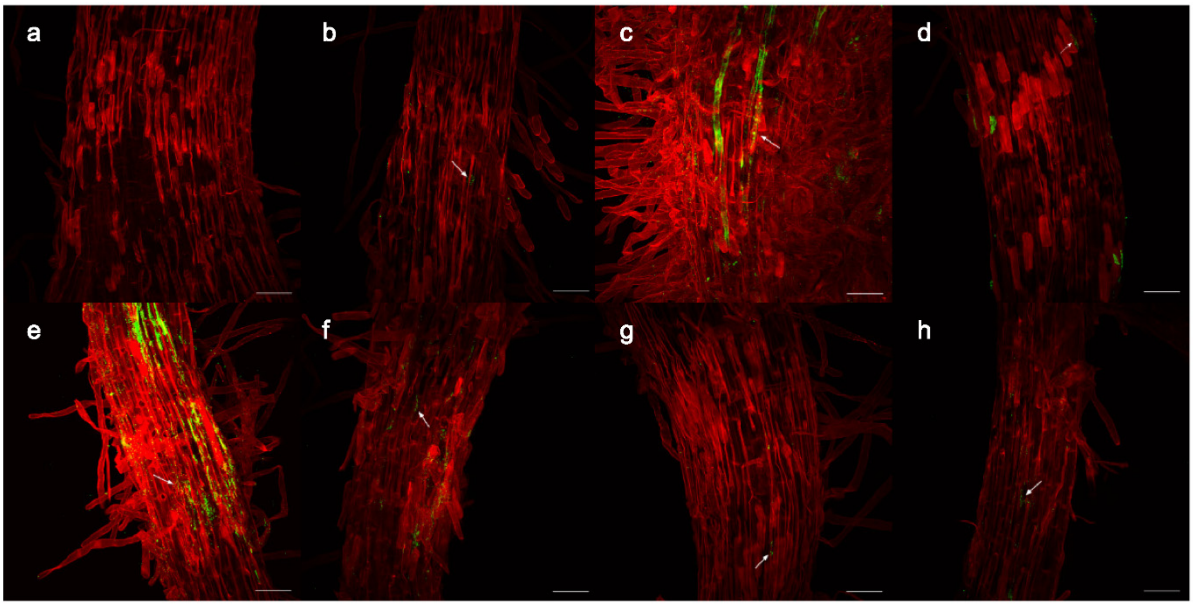

Table S1. The sample size for each bacterial treatment and each plant genotype. The number of plant individuals (the number of replicates in benign water conditions / number of replicates in drought conditions) is given. Names of bacterial strains are provided in the Materials and Methods.

Plant genotype	Bacterial treatment							
	Control	YJ1	YJ4	YJ6	SD1	MS26	GG1	GG19
P1	2 / 3	2 / 4	2 / 4	2 / 4	2 / 4	2 / 4	2 / 4	2 / 4
P2	2 / 3	2 / 4	2 / 4	2 / 4	2 / 4	2 / 4	2 / 4	2 / 4
P3	2 / 2	2 / 4	2 / 4	2 / 4	2 / 4	2 / 4	2 / 4	2 / 4
P4	2 / 3	2 / 4	2 / 4	2 / 4	2 / 4	2 / 4	2 / 4	2 / 4
P5	2 / 4	2 / 4	2 / 4	2 / 4	2 / 4	2 / 4	2 / 4	2 / 4
Total	10 /15	10 / 20	10 / 20	10 / 20	10 / 20	10 / 20	10 / 20	10 / 20
